# Marital status and ischemic heart disease incidence and mortality in women: a large prospective study

**DOI:** 10.1186/1741-7015-12-42

**Published:** 2014-03-12

**Authors:** Sarah Floud, Angela Balkwill, Dexter Canoy, F Lucy Wright, Gillian K Reeves, Jane Green, Valerie Beral, Benjamin J Cairns

**Affiliations:** 1 Cancer Epidemiology Unit, University of Oxford, Roosevelt Drive, Oxford OX3 7LF, UK

**Keywords:** Marital status, Ischemic heart disease, Incidence, Mortality, Women

## Abstract

**Background:**

Being married has been associated with a lower mortality from ischemic heart disease (IHD) in men, but there is less evidence of an association for women, and it is unclear whether the associations with being married are similar for incident and for fatal IHD. We examined the relation between marital status and IHD incidence and mortality in the Million Women Study.

**Methods:**

A total of 734,626 women (mean age 60 years) without previous heart disease, stroke or cancer, were followed prospectively for hospital admissions and deaths. Adjusted relative risks (RRs) for IHD were calculated using Cox regression in women who were married or living with a partner versus women who were not. The role of 14 socio-economic, lifestyle and other potential confounding factors was investigated.

**Results:**

81% of women reported being married or living with a partner and they were less likely to live in deprived areas, to smoke or be physically inactive, but had a higher alcohol intake than women who were not married or living with a partner. During 8.8 years of follow-up, 30,747 women had a first IHD event (hospital admission or death) and 2,148 died from IHD. Women who were married or living with a partner had a similar risk of a first IHD event as women who were not (RR = 0.99, 95% confidence interval (CI) 0.96 to 1.02), but a significantly lower risk of IHD mortality (RR = 0.72, 95% CI 0.66 to 0.80, *P* <0.0001). This lower risk of IHD death was evident both in women with and without a prior IHD hospital admission (respectively: RR = 0.72, 95% CI 0.60 to 0.85, *P* <0.0001, n = 683; and 0.70, 95% CI 0.62 to 0.78, *P* <0.0001, n = 1,465). These findings did not vary appreciably between women of different socio-economic groups or by lifestyle and other factors.

**Conclusions:**

After adjustment for socioeconomic, lifestyle and other factors, women who were married or living with a partner had a similar risk of developing IHD but a substantially lower IHD mortality compared to women who were not married or living with a partner.

## Background

Studies conducted over several decades and on different populations have shown that being married is associated with a lower risk of all-cause mortality [1-3]. Being married has also been associated with a lower risk of ischemic heart disease (IHD) mortality in men [4-8], but in women any reported lower risk of IHD has not been statistically significant [6,8]. It may be that the presence of a spouse influences prognosis after the onset of IHD through encouragement to seek early medical attention for symptoms or to comply with a treatment regime [9,10]. It has also been proposed that being married may protect against developing disease by encouraging a healthier lifestyle [11,12] or by providing social support [13,14] or financial security [11], but the published data do not clearly show whether being married influences the onset of IHD for either men [7,15-17] or women [16,17].

We investigated the association of marital status with IHD incidence and mortality in a large prospective cohort of middle-aged women in the UK. We also examined the risk of IHD death in women after a first hospital admission for IHD, and the extent to which socio-economic, lifestyle and other factors might explain any association between marital status and IHD incidence or mortality.

## Methods

### Study design and participants

Between 1996 and 2001, 1.3 million women were recruited to the Million Women Study via the UK national breast screening program [18]. After an average of three years, these women were resurveyed using a new postal questionnaire, with a response rate of 65%. In the resurvey, participants were asked for the first time about their marital status, and the date of the resurvey is, therefore, the baseline study date for the current analysis. The full study questionnaires are available at [19]. The respondents gave written consent to participate and ethics approval was provided by the Oxford and Anglia Multi-Centre Research Ethics Committee. The follow-up is virtually complete for deaths, cancers and hospital admissions because all participants are linked by their unique National Health Service (NHS) identification number to NHS Central Registers, through which they are followed for death, emigration and cancer registration, and to the NHS hospital admissions databases. Information on the date of admission and discharge and diagnoses associated with each hospital admission, coded to the World Health Organization’s International Classification of Diseases 10^th^ revision (ICD-10) [20], was obtained by electronic record linkage to the Hospital Episode Statistics for England (HES) [21] and Scottish Morbidity Records in Scotland [22].

### Marital status and covariates

Marital status at baseline was assessed by asking “Are you currently married or living with a partner?” Those who replied “yes” are referred to as partnered and those who did not are referred to as unpartnered. The unpartnered category thus includes women who were never married, as well as women who were divorced, separated or widowed. It is likely that the vast majority of the partnered category were married and that a large proportion of the unpartnered category were divorced, separated or widowed, since the General Household Survey for 2002 reported that 71% of women aged 55 to 64 years old were married, 3% were cohabiting, 4% were single, 13% were divorced or separated and 9% were widowed [23]. We compared marital status at baseline with marital status reported at the next resurvey, an average of 4.5 years (SD: 1.2 years) later, and found excellent agreement: 94% of women partnered at baseline again reported being partnered, and 94% of unpartnered women again reported being unpartnered (kappa statistic for agreement = 0.81). Therefore, we used marital status at baseline in our analyses. We also compared marital status at baseline with reports of how many people lived in their household nine years later. Only 12% of women who were partnered at baseline reported nine years later that they were living alone compared to 79% of the unpartnered women.

Socio-economic status was measured at recruitment and assessed using quintiles of the Townsend area deprivation score [24] and two measures of education: highest qualification (O levels, A levels, Nursing/Teaching, College/University, none of the preceding categories) and age at leaving school (left school before or at the compulsory school leaving age, left school after the compulsory school leaving age, no schooling). This latter variable took into account the change in the compulsory school leaving age from 14 to 15 which occurred on 1 April 1947 in both England [25] and Scotland [26].

The lifestyle risk factors assessed were cigarette smoking (never, past, current <15 per day, current ≥15 per day), alcohol intake (0, <7, 7 to 14, ≥15 drinks per week), strenuous exercise (rarely or never, <once per week, ≥once per week), body mass index (BMI) (<22.5, 22.5 to 24.9, 25.0 to 27.4, 27.5 to 29.9, ≥30 kg/m^2^), sleep duration (<7, 7, 8, ≥9 hours) and hormone replacement therapy use (never, ever). These variables were recorded at baseline, except strenuous exercise, which was recorded at recruitment.

Other factors assessed were two measures of well-being: reported happiness at baseline (rarely/never, sometimes, usually, most of the time) and treatment for depression reported at recruitment or baseline (yes, no). In addition, three measures which reflected social contact were assessed: parity recorded at recruitment (nulliparous, parous), current employment at baseline (not in paid work, part-time, full-time) and participation in group activities, such as religious group, voluntary work, art/craft class, sports club, dancing group, music group, bingo, yoga and other group activity, at baseline (none, one, two, three or more group activities).

### Ischemic heart disease

A first IHD event was defined as a first hospital admission for IHD or death with IHD as the underlying cause. The definition of a hospital admission for IHD was any mention of an IHD diagnosis (ICD-10: I20 to I25) in a primary or other diagnosis field in the hospital record. In a study of vascular disease outcomes in this cohort, IHD information based on hospital records and general practice records were consistent in 92% of 796 randomly selected women with a hospital record of IHD [27]. IHD mortality was defined as death with IHD as the underlying cause (ICD-10: I20 to I25) at any point during follow-up, with or without a prior hospital admission. First IHD events were also subdivided into: (i) death from IHD with no prior hospital admission and (ii) first hospital admission for IHD. The small number of women (n = 76) who died on the day of their first hospital admission for IHD were classed as IHD deaths.

### Analysis

A total of 866,334 women completed the baseline questionnaire. We excluded 74,693 (8.6%) women who reported heart disease or stroke or had been admitted to a hospital for these conditions, and 42,827 (4.9%) women who had a cancer registration (except non-melanoma skin cancer), prior to baseline. A further 14,188 (1.6%) women were excluded for whom information on marital status was missing. The remaining 734,626 women formed the population at risk for these analyses.

We used Cox regression to estimate relative risks (RR) and 95% confidence intervals (CI) of first IHD events and IHD mortality. Relative risks were also estimated separately for: IHD death without prior hospital admission; first IHD hospital admission; and IHD death after hospital admission. Person-years were calculated from baseline until the date of hospital admission for IHD, death, emigration or end of follow-up, whichever came first. Women were followed until 31 March 2011 in England and 31 December 2008 in Scotland (7% of women in analysis lived in Scotland), because complete hospital admission data were not available after these dates.

The regression models used attained age as the underlying time variable and were stratified by region of residence at recruitment (Scotland, and nine regions in England) and adjusted separately and simultaneously for three groups of covariates: (i) indicators of socio-economic status, (ii) lifestyle risk factors and (iii) other factors. Missing data for the adjustment variables (<2.1% for each variable) were assigned to a separate category. Heterogeneity in the associations between marital status and first IHD events or IHD mortality by sub-groups of age, region and socio-economic, lifestyle and other factors, was assessed using a chi-squared contrast test [28].

For risk of IHD death after hospital admission for IHD, person-years at risk were calculated from first hospital admission for IHD to death, emigration or end of follow-up. Any difference in risks of IHD death associated with marital status during the hospital stay and after discharge was investigated by splitting the follow-up period at 28 days after first hospital admission.

To assess the possibility of reverse causation, where early symptoms of disease might affect the likelihood of marriage breakdown [29], we conducted two sensitivity analyses. In one sensitivity analysis, we excluded the first five years of follow-up and, in the other, we restricted the analysis to women who rated their health as “good” or “excellent” at baseline. All analyses used Stata 12.1 (StataCorp., College Station, TX, USA).

## Results

At baseline, the mean age of the women was 59.7 years (SD: 4.8 years); 81% reported being married or living with a partner (partnered) (Table 1). The main differences between partnered and unpartnered women were that partnered women were less likely to live in deprived areas, to smoke, or to be physically inactive, but there was little difference in mean BMI, and partnered women had a slightly higher intake of alcohol (Table 1). Partnered women were also less likely to report that they had been treated for depression or that they were rarely, never or (only) sometimes happy. They were more likely to be employed than unpartnered women but less likely to report participation in group activities.

**Table 1:** Characteristics and details of follow-up for ischemic heart disease, by marital status

	**Marital status**		
	**Partnered**	**Unpartnered**	**All Women**
**Characteristics**^**a**^	n = 594,675 (81%)	n = 139,951 (19%)	n = 734,626 (100%)
Mean age, years (SD)	59.5 (4.7)	60.8 (5.2)	59.7 (4.8)
**Socio-economic factors:**			
Most deprived quintile,%	14.0	24.4	16.0
Left school ≤ minimum leaving age,%	48.4	47.0	48.1
No educational qualifications,%	49.0	46.3	48.5
**Lifestyle factors:**			
Current smoker,%	11.1	17.1	12.2
Mean alcohol, drinks/week (SD)	4.7 (5.8)	3.7 (5.6)	4.5 (5.8)
Strenuous exercise rarely/never,%	43.0	45.5	43.5
Mean body mass index, kg/m^2^ (SD)	26.0 (4.4)	26.1 (4.9)	26.0 (4.5)
Never users of HRT,%	45.1	50.9	46.2
Mean number of hours asleep (SD)	7.3 (1.1)	7.2 (1.3)	7.3 (1.2)
**Other factors:**			
Rarely/never/sometimes happy,%	15.2	23.7	16.8
Treatment for depression,%	9.1	14.9	10.2
Mean number of children (SD)	2.1 (1.1)	1.9 (1.4)	2.1 (1.2)
Not in work,%	52.9	56.1	53.5
No participation in group activities,%	36.4	32.8	35.7
**Follow-up for IHD incidence and mortality (I20 to I25)**			
Mean years of follow-up (SD)	8.8 (1.9)	8.6 (2.0)	8.8 (1.9)
First IHD event, n	23,816	6,931	30,747
IHD deaths^b^, n	974	491	1,465
First hospital admissions for IHD, n	22,842	6,440	29,282
All IHD deaths, n	1,442	706	2,148

^a^Percentages were calculated based on women with complete information for that specific variable.

^b^IHD deaths without prior IHD hospital admission.

IHD, ischemic heart disease; HRT, hormone replacement therapy.

**Figure 1:**
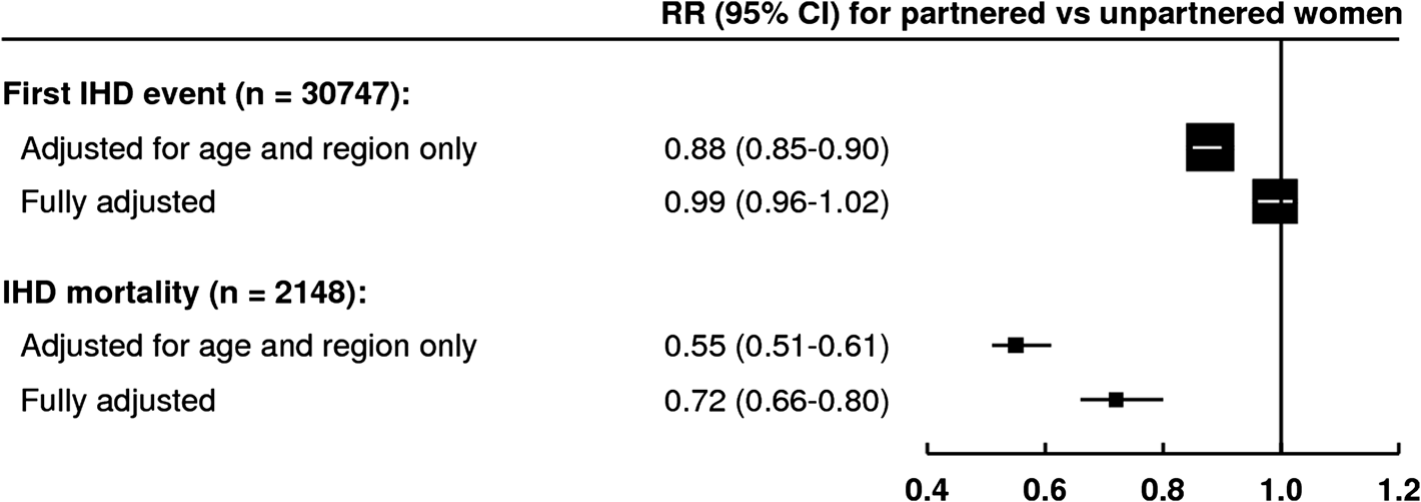
**Relative risk of ischemic heart disease first event and mortality in relation to marital status.** Relative risks (RRs) presented with 95% confidence intervals (95% CI). Fully adjusted for: age, region, area deprivation, age left school, highest educational qualification, smoking, alcohol intake, strenuous exercise, body mass index, hormone replacement therapy use, sleep duration, happiness, treatment for depression, parity, employment and participation in group activities. IHD, ischemic heart disease.

During an average follow-up of 8.8 years per woman, there were 30,747 first IHD events (including 29,282 hospital admissions for IHD, and 1,465 deaths without prior hospital admission) and, overall, 2,148 women died of IHD (Table 1). With minimal adjustment for age and region of recruitment only, partnered women had a lower risk of a first IHD event and lower IHD mortality than unpartnered women, but adjustment for lifestyle risk factors, particularly smoking and area deprivation attenuated the risk estimates (see Additional file 1: Table S1). After adjustment for all socioeconomic, lifestyle and other risk factors, partnered women had a similar risk of a first IHD event as unpartnered women (adjusted RR = 0.99, 95% CI 0.96 to 1.02) but had significantly lower IHD mortality (adjusted RR = 0.72, 95% CI 0.66 to 0.80, *P* <0.0001) (Figure 1).

When first IHD events were subdivided into whether the event was a hospital admission or a death, partnered women had a similar risk of first hospital admission for IHD as unpartnered women (adjusted RR = 1.01, 95% CI 0.98 to 1.04) but significantly lower risk of death from IHD with no prior hospital admission (adjusted RR = 0.70, 95% CI 0.62 to 0.78, *P* <0.0001) (Table 2).

**Table 2:** Relative risk of ischemic heart disease first event and mortality comparing partnered to unpartnered women

	**First IHD event**	**IHD mortality**	**Subdivisions of first IHD event**		**Survival**
	**IHD hospital admission or IHD death**	**All IHD deaths**	**IHD death with no prior hospital admission**	**IHD hospital admission**	**IHD death after hospital admission**
**Population at risk (n)**	734,626	734,626	734,626	734,626	29,282
**Cases (n)**	30,747	2,148	1,465	29,282	683
	RR (95% CI)	RR (95% CI)	RR (95% CI)	RR (95% CI)	RR (95% CI)
**Adjusted for age and region only**	**0.88 (0.85 to 0.90)**	**0.55 (0.51 to 0.61)**	**0.53 (0.47 to 0.59)**	**0.90 (0.88 to 0.93)**	**0.61 (0.52 to 0.72)**
**Additionally adjusted only for socio-economic factors**^**a**^	0.91 (0.89 to 0.94)	0.59 (0.54 to 0.65)	0.57 (0.51 to 0.64)	0.94 (0.91 to 0.96)	0.63 (0.54 to 0.75)
**Additionally adjusted only for lifestyle factors**^**b**^	0.96 (0.94 to 0.99)	0.68 (0.62 to 0.74)	0.65 (0.58 to 0.72)	0.99 (0.96 to 1.02)	0.70 (0.59 to 0.82)
**Additionally adjusted only for other factors**^**c**^	0.88 (0.86 to 0.91)	0.56 (0.51 to 0.62)	0.54 (0.49 to 0.61)	0.91 (0.88 to 0.93)	0.62 (0.53 to 0.74)
**Adjusted for all the above**^**d**^	**0.99 (0.96 to 1.02)**	**0.72 (0.66 to 0.80)**	**0.70 (0.62 to 0.78)**	**1.01 (0.98 to 1.04)**	**0.72 (0.60 to 0.85)**

^a^adjusted for age, region, area deprivation, age left school, highest educational qualification.

^b^adjusted for age, region, smoking, alcohol intake, strenuous exercise, body mass index, hormone replacement therapy use, sleep duration.

^c^adjusted for age, region, happiness, treatment for depression, parity, employment, participation in group activities.

^d^fully adjusted for age, region, area deprivation, age left school, highest educational qualification, smoking, alcohol intake, strenuous exercise, body mass index, hormone replacement therapy use, sleep duration, happiness, treatment for depression, parity, employment, participation in group activities.

CI, confidence interval; IHD, ischemic heart disease; RR, relative risk.

**Figure 2:**
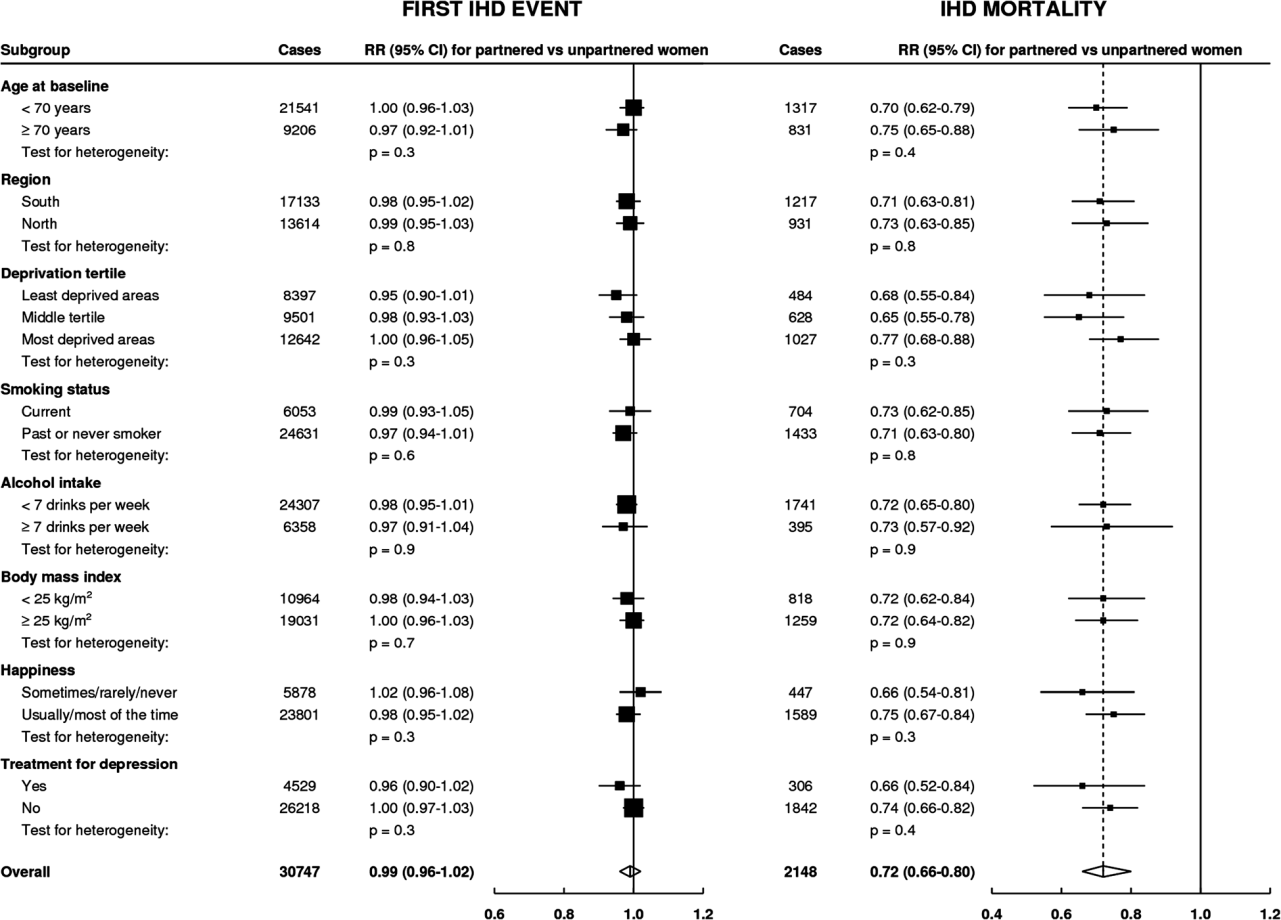
**Relative risk of ischemic heart disease first event and mortality by marital status, in subgroups.** Relative risks (RRs) presented with 95% confidence intervals (95% CI). The dotted line represents the RR of IHD mortality for all women, comparing partnered to unpartnered. RRs are adjusted as appropriate for age, region, area deprivation, age left school, highest educational qualification, smoking, alcohol intake, strenuous exercise, body mass index, hormone replacement therapy use, sleep duration, happiness, treatment for depression, parity, employment and participation in group activities. IHD, ischemic heart disease.

The findings did not differ materially by subgroups of age, region or level of area deprivation, by lifestyle factors, such as smoking, alcohol intake and body mass index, or by measures of well-being, such as happiness and treatment for depression (Figure 2). There was no evidence of heterogeneity across subgroups of the remaining factors (age left school, strenuous activity, sleep duration, HRT use, parity, employment, participation in group activities), except for weak evidence of a difference for first IHD events by whether the women were in paid work or not; this difference could have arisen by chance, due to the large number of significance tests performed (see Additional file 1: Figure S1). The risk estimates were not materially changed when we excluded the first five years of follow-up (see Additional file 1: Table S2) or when we restricted the analysis to women who rated their health as “good” or “excellent” (see Additional file 1: Table S3).

Among the 29,282 women who had a first hospital admission for IHD, the relationships between marital status and socioeconomic, lifestyle and other characteristics were similar to those found in the main sample (see Additional file 1: Table S4). When their survival was examined over a mean follow-up period of 3.7 years per woman, partnered women were less likely than unpartnered women to die from IHD after their first hospital admission for IHD (adjusted RR = 0.72, 95% CI 0.60 to 0.85; n = 683) (Table 2). The lower risks for partnered women were evident both in the first 28 days following a hospital admission, and in later follow-up (respective adjusted RRs: 0.74, 95% CI 0.57 to 0.98, n = 350; and 0.69, 95% CI 0.54 to 0.89, n = 333). Among women who had a first hospital admission for IHD, we examined use of common medications for IHD, reported two years later on average, and found little difference in the pattern of use between partnered and unpartnered women (68% of partnered women reported using statins vs 68% of unpartnered women; using diuretics 27% vs 27%; and using beta-blockers 35% vs 33%).

## Discussion

In this large prospective cohort of middle-aged UK women, women who were married or living with a partner had similar risks of a first IHD event as women who were not married or living with a partner, after adjustment for socio-economic, lifestyle and other risk factors. In contrast, women who were married or living with a partner were at lower risk of IHD mortality and this lower risk remained after adjustment for the same factors, and was found in women both with and without a prior hospital admission for IHD. Unlike previous studies, the large sample size of the Million Women Study cohort allowed us to investigate whether the associations between marital status and IHD differed across a range of subgroups of socio-economic, lifestyle and other factors. After accounting for the multiplicity of statistical tests, we found that there was little evidence that the associations varied between subgroups of these factors.

To our knowledge, this is the first study of women to investigate the effect of marital status on both IHD incidence and mortality within the same cohort, although our finding of a lower risk for IHD mortality, but not incidence, has also been reported in men [7,15]. The previous evidence on incident IHD events in women in relation to marital status is sparse. Two previous cohort studies have examined the association between marital status and incident IHD in women. A population-based cohort study in Sweden with 507 incident IHD events reported no significant difference in risk by marital status [16], but a recent register-based study in Finland, with 7,193 IHD events, reported a lower risk for married women, but did not adjust for socio-economic or lifestyle risk factors [17].

In our study, the association between marital status and incident IHD was attenuated after adjustment for area deprivation and lifestyle risk factors, which suggests that any influence of marital status on the development of IHD may be confounded with or mediated through these factors. Methodologically, it is difficult to distinguish between factors which may be confounders of the association and those which may be mediators. Marital status has been proposed to influence risk factors for IHD in several ways. For example, spousal influences on behavior may encourage healthier lifestyles [11,12], or there may be negative changes in lifestyle after divorce or separation [30,31]. However, people may choose partners who share their behaviors and, therefore, marriage or cohabitation may reinforce both beneficial and harmful lifestyle choices. Area deprivation might act as another mediator, given that getting married can enhance one’s financial resources, whereas divorce or widowhood can have the reverse effect [11]. It was not possible to adjust further for individual-level measures of deprivation, since information on household income was not collected. Social support has also been proposed to mediate the association between marital status and health [13,14,32], but in this study adjustment for variables which could indicate social interaction, including parity, participation in group activities and employment, and measures of well-being, such as reported happiness and treatment for depression, had little effect on the risk estimates. However, we cannot exclude possible roles of unmeasured aspects of social support, such as the frequency of social contact or the quality of social support.

There is little previous evidence on IHD mortality in relation to marital status in women in the general population. Being married has been associated with lower risks of overall cardiovascular mortality in women [3,8,33], but these associations could be driven by common vascular diseases other than IHD, such as stroke and venous thromboembolism. There have been two cohort studies that have reported on mortality from IHD but they included relatively few women and reported no significant difference in risk between married and unmarried women [6,8]. We found lower risks of IHD death in partnered women with no prior hospital admission, consistent with evidence that being married or cohabiting is associated with lower risks of out-of-hospital sudden cardiac arrest [34], pre-hospital deaths from myocardial infarction [17] and lower case fatality rates for first day of a coronary event [16]. We also found lower risks of IHD mortality in partnered women after a hospital admission for IHD. This fits with evidence from smaller patient populations (of up to 1,500 patients) in which there were higher risks of deaths following hospitalization for IHD for non-married patients or those living alone [35-39], although two larger studies (up to 16,000 patients) did not find a higher risk of IHD death associated with living alone [40,41].

The lower risk of IHD mortality for partnered women in our study was only partly attenuated after all adjustments, suggesting that marital status influences IHD mortality in part by modifying a woman’s response to the disease, although residual confounding cannot be ruled out. In this cohort, unpartnered women tended to live alone, so a possible explanation for the lower risk of death among partnered women may be that they have someone at home who can respond to symptoms and help them seek appropriate treatment [9,42]. Spouses have been shown to encourage their partners to comply with effective medication regimes [43], facilitate attendance at cardiac rehabilitation programs [10], and support modification of lifestyle risk factors [30,44]. However, the information available to us on medication use does not support a greater level of compliance in partnered women compared to unpartnered women. Spouses can also provide emotional support to cope with the distress of having had a cardiac event [14]. Another explanation for the improved survival after hospital admission among partnered women is that they may tend to have less severe disease on admission to hospital, but we were unable to assess this due to lack of data on disease severity.

Marital status itself was relatively stable during follow-up in this study, but we do not know if the women who were unpartnered at baseline were never married, divorced, separated or widowed, although the 2002 General Household Survey indicated that most would be divorced, separated or widowed [23]. This unpartnered category is therefore diverse and it could be that being divorced or widowed rather than never married places women at higher risk of IHD, but findings from previous cohort studies show little consistency in the associations between IHD mortality and the various non-married states for women [8,16,45]. It is possible that healthy women may be less likely to divorce [29]. However, we were able to limit bias associated with this by excluding women with pre-existing disease, and also through two sensitivity analyses that showed no material change in the adjusted risk estimates.

## Conclusions

In this large UK cohort of middle-aged women, being married or living with a partner does not appear to affect the risk of developing IHD after adjustment for socioeconomic, lifestyle and other factors. However, there remains a substantial, unexplained lower risk of death from IHD for women who are married or living with a partner compared to women who are not.

## Abbreviations

BMI: Body mass index; ICD-10: International Statistical Classification of Disease and Related Health Problems: 10^th^ Revision; IHD: Ischemic heart disease; HRT: hormone replacement therapy; NHS: National Health Service.

## Competing interests

The authors declare that they have no competing interests.

## Authors’ contributions

VB, GKR and JG were involved in the conception, design and data acquisition for the Million Women Study. SF, BJC, AB and VB analyzed and interpreted the data. SF drafted the first version of the manuscript. All authors contributed to drafting revised versions of the manuscript and gave their final approval of the version to be published.

## Pre-publication history

The pre-publication history for this paper can be accessed here:

http://www.biomedcentral.com/1741-7015/12/42/prepub

## Supplementary Material

Additional file 1: Table S1 Relative risk of ischemic heart disease first event and mortality comparing partnered to unpartnered women, with separate adjustments for various characteristics. **Figure S1.** Relative risk of ischemic heart disease first event and mortality comparing partnered to unpartnered women, within further subgroups. **Table S2.** Relative risk of ischemic heart disease first event and mortality comparing partnered to unpartnered women, excluding first five years of follow-up. **Table S3.** Relative risk of ischemic heart disease first event and mortality comparing partnered to unpartnered women, restricted to women who rated their health as “good” or “excellent” at baseline. **Table S4.** Characteristics and details of follow-up for ischemic heart disease (IHD) mortality in the subsample of women whose first event was a hospital admission for IHD, by marital status. Click here for file
