# Supplementary material for: Marital status and ischemic heart disease incidence and mortality in women: a large prospective study
Source: BMC Med. 2014 Mar 12;12:42. doi: 10.1186/1741-7015-12-42 (PMC4103700; doi:10.1186/1741-7015-12-42)
Supplement: Additional file 1: Table S1 — Relative risk of ischemic heart disease first event and mortality comparing partnered to unpartnered women, with separate adjustments for various characteristics. Figure S1. Relative risk of ischemic heart disease first event and mortality comparing partnered to unpartnered women, within further subgroups. Table S2. Relative risk of ischemic heart disease first event and mortality comparing partnered to unpartnered women, excluding first five years of follow-up. Table S3. Relative risk of ischemic heart disease first event and mortality comparing partnered to unpartnered women, restricted to women who rated their health as “good” or “excellent” at baseline. Table S4. Characteristics and details of follow-up for ischemic heart disease (IHD) mortality in the subsample of women whose first event was a hospital admission for IHD, by marital status. [file 1741-7015-12-42-S1.pdf]

ADDITIONAL FILE 1

Marital status and ischemic heart disease incidence and mortality in women: a large prospective study

Sarah Floud[1], Angela Balkwill[1], Dexter Canoy[1], F Lucy Wright[1], Gillian K Reeves[1], Jane Green[1], Valerie Beral[1], Benjamin J Cairns[1], for the Million Women Study Collaborators

1: Cancer Epidemiology Unit, University of Oxford, Roosevelt Drive, Oxford, OX3 7LF

Table S1 Relative risk of ischemic heart disease first event and mortality comparing partnered to unpartnered women, with separate adjustments for various characteristics

	FIRST IHD EVENT	IHD MORTALITY
	IHD hospital admission or IHD death	All IHD deaths
**Population at risk (n)**	734,626	734,626
**Cases (n)**	30,747	2,148
	RR (95% CI)	RR (95% CI)
**Adjusted for age and region only**	**0.88 (0.85-0.90)**	**0.55 (0.51-0.61)**
**Additional adjustment separately for:**		
**socio-economic factors:**		
area deprivation	0.94 (0.91-0.96)	0.61 (0.56-0.67)
age left school	0.87 (0.85-0.89)	0.55 (0.50-0.60)
qualification	0.87 (0.84-0.89)	0.54 (0.49-0.60)
**lifestyle factors:**		
smoking	0.93 (0.91-0.96)	0.63 (0.58-0.70)
alcohol intake	0.91 (0.89-0.94)	0.59 (0.53-0.64)
strenuous exercise	0.88 (0.86-0.91)	0.56 (0.51-0.61)
body mass index	0.88 (0.86-0.91)	0.56 (0.51-0.62)
hormone replacement therapy use	0.87 (0.85-0.89)	0.56 (0.52-0.62)
sleep duration	0.89 (0.86-0.91)	0.55 (0.51-0.61)
**other factors:**		
happiness	0.90 (0.88, 0.93)	0.58 (0.52-0.63)
depression	0.90 (0.88-0.93)	0.57 (0.52-0.62)
parity	0.86 (0.83-0.88)	0.57 (0.52-0.62)
employment	0.88 (0.85-0.90)	0.55 (0.50-0.60)
participation in group activities	0.87 (0.84-0.89)	0.53 (0.49-0.59)

Figure S1 Relative risk of ischemic heart disease first event and mortality comparing partnered to unpartnered women, within further subgroups.

Subgroup	FIRST IHD EVENT Cases	FIRST IHD EVENT RR (95% CI) for partnered vs unpartnered women	IHD MORTALITY Cases	IHD MORTALITY RR (95% CI) for partnered vs unpartnered women
**Age left school**				
≤ Compulsory leaving age	18348	1.01 (0.97-1.04)	1303	0.75 (0.67-0.85)
≥ Compulsory leaving age	12271	0.97 (0.92-1.01)	836	0.67 (0.57-0.78)
Test for heterogeneity:		p = 0.2		p = 0.2
**Strenuous activity**				
< Once per week	19260	1.00 (0.97-1.04)	1450	0.70 (0.63-0.79)
≥ Once per week	10448	0.96 (0.92-1.01)	602	0.78 (0.65-0.93)
Test for heterogeneity:		p = 0.2		p = 0.4
**Sleep**				
< 8 hours	17226	1.00 (0.97-1.04)	1120	0.78 (0.69-0.89)
≥ 8 hours	13115	0.96 (0.92-1.00)	989	0.66 (0.57-0.76)
Test for heterogeneity:		p = 0.2		p = 0.1
**HRT use**				
Never	13316	0.97 (0.93-1.01)	1259	0.69 (0.61-0.78)
Ever	16679	1.01 (0.97-1.05)	817	0.77 (0.66-0.91)
Test for heterogeneity:		p = 0.1		p = 0.3
**Parity**				
Nulliparous	2958	0.99 (0.91-1.07)	310	0.65 (0.51-0.82)
Parous	27737	0.99 (0.96-1.02)	1835	0.74 (0.67-0.82)
Test for heterogeneity:		p = 0.9		p = 0.3
**Employment**				
Not in paid work	20250	0.97 (0.93-1.00)	1609	0.71 (0.64-0.79)
In paid work	10087	1.04 (0.99-1.09)	510	0.76 (0.62-0.93)
Test for heterogeneity:		p = 0.02		p = 0.6
**Participation in group activities**				
No	12060	0.97 (0.93-1.02)	996	0.71 (0.62-0.82)
Yes	18687	1.00 (0.97-1.04)	1152	0.75 (0.65-0.85)
Test for heterogeneity:		p = 0.3		p = 0.6
**Overall**	**30747**	**0.99 (0.96-1.02)**	**2148**	**0.72 (0.66-0.80)**

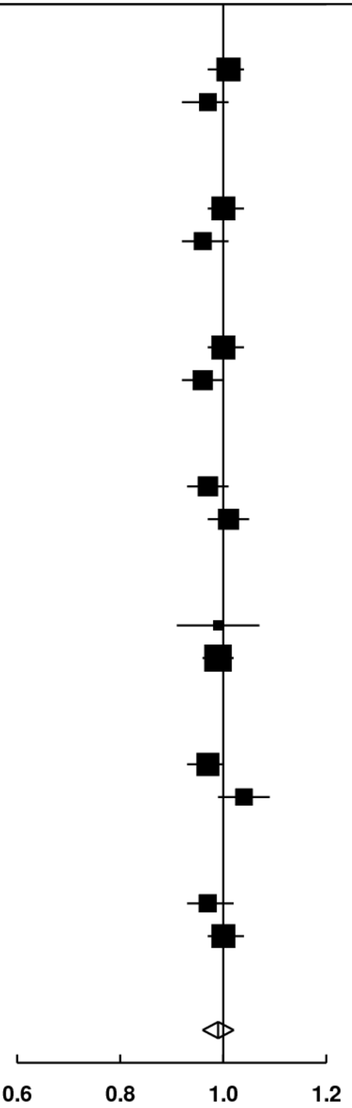

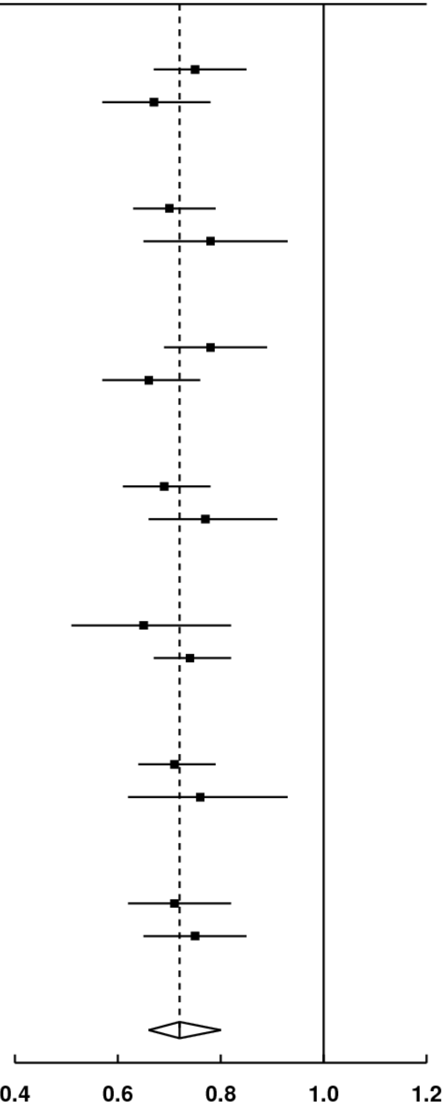

The dotted line represents the relative risks (RR) of IHD mortality for all women, comparing partnered to unpartnered. RRs are adjusted as appropriate for age, region, area deprivation, age left school, highest educational qualification, smoking, alcohol intake, strenuous exercise, body mass index, sleep duration, hormone replacement therapy use, happiness, treatment for depression, parity, employment, participation in group activities. Abbreviations: CI, confidence interval; IHD, ischemic heart disease; HRT, hormone replacement therapy; RR, relative risk

Table S2 Relative risk of ischemic heart disease first event and mortality comparing partnered to unpartnered women, excluding first five years of follow-up

	**FIRST IHD EVENT**	**IHD MORTALITY**
	IHD hospital admission or IHD death	All IHD deaths
**Population at risk (n)**	700,736[a]	713,529[a]
**Cases (n)**	16,294	1,180
	RR (95% CI)	RR (95% CI)
**Adjusted for age and region only**	**0.88 (0.84-0.91)**	**0.58 (0.51-0.66)**
**Additionally adjusted only for socio-economic factors[b]**	0.91 (0.87-0.94)	0.62 (0.54-0.70)
**Additionally adjusted only for lifestyle factors[c]**	0.96 (0.92-0.99)	0.71 (0.62-0.80)
**Additionally adjusted only for other factors[d]**	0.88 (0.84-0.91)	0.59 (0.52-0.67)
**Adjusted for all the above[e]**	**0.97 (0.94-1.01)**	**0.75 (0.66-0.86)**

[a] populations at risk are not the same for first IHD event analysis and IHD mortality analysis because in the mortality analysis we excluded women who died in the first five years, whereas in the analysis for first IHD event we excluded both women who died in the first five years and women who had a first hospital admission for IHD.
[b] adjusted for age, region, area deprivation, age left school, highest educational qualification
[c] adjusted for age, region, smoking, alcohol intake, strenuous exercise, body mass index, hormone replacement therapy use, sleep duration
[d] adjusted for age, region, happiness, treatment for depression, parity, employment, participation in group activities
[e] fully adjusted for age, region, area deprivation, age left school, highest educational qualification, smoking, alcohol intake, strenuous exercise, body mass index, hormone replacement therapy use, sleep duration, happiness, treatment for depression, parity, employment, participation in group activities
Abbreviations: CI, confidence interval; IHD, ischemic heart disease; RR, relative risk

Table S3 Relative risk of ischemic heart disease first event and mortality comparing partnered to unpartnered women, restricted to women who rated their health as “good” or “excellent” at baseline

	**FIRST IHD EVENT**	**IHD MORTALITY**
	IHD hospital admission or IHD death	All IHD deaths
**Population at risk (n)**	569475	569475
**Cases (n)**	18862	1166
	RR (95% CI)	RR (95% CI)
**Adjusted for age and region only**	**0.95 (0.92, 0.98)**	**0.61 (0.54, 0.70)**
**Additionally adjusted only for socio-economic factors**[b]	0.97 (0.93, 1.00)	0.64 (0.56, 0.73)
**Additionally adjusted only for lifestyle factors**[c]	1.02 (0.98, 1.06)	0.73 (0.64, 0.83)
**Additionally adjusted only for other factors**[d]	0.93 (0.90, 0.97)	0.60 (0.53, 0.69)
**Adjusted for all the above**[e]	**1.02 (0.98, 1.06)**	**0.76 (0.66, 0.87)**

[b] adjusted for age, region, area deprivation, age left school, highest educational qualification
[c] adjusted for age, region, smoking, alcohol intake, strenuous exercise, body mass index, hormone replacement therapy use, sleep duration
[d] adjusted for age, region, happiness, treatment for depression, parity, employment, participation in group activities
[e] fully adjusted for age, region, area deprivation, age left school, highest educational qualification, smoking, alcohol intake, strenuous exercise, body mass index, hormone replacement therapy use, sleep duration, happiness, treatment for depression, parity, employment, participation in group activities
Abbreviations: CI, confidence interval; IHD, ischemic heart disease; RR, relative risk

Table S4 Characteristics and details of follow-up for ischemic heart disease (IHD) mortality in the subsample of women whose first event was a hospital admission for IHD, by marital status

	**Marital status**		
	**Partnered**	**Unpartnered**	**All Women**
**Characteristics**[a]	n = 22,842 (78%)	n = 6,440 (22%)	n = 29,282 (100%)
Mean age, years (SD)	61.1 (4.9)	62.6 (5.4)	61.5 (5.1)
**Socio-economic factors:**			
Most deprived quintile, %	19.8	30.8	22.2
Left school ≤ minimum leaving age, %	60.5	57.8	59.9
No educational qualifications, %	59.5	56.8	58.9
**Lifestyle factors:**			
Current smoker, %	17.0	23.5	18.4
Mean alcohol, drinks/week (SD)	3.7 (5.4)	2.9 (5.1)	3.5 (5.3)
Strenuous exercise rarely/never, %	52.0	53.2	52.2
Mean body mass index, kg/m^2 (SD)	27.0 (4.8)	27.1 (5.4)	27.0 (4.9)
Never users of hormone replacement therapy, %	42.1	50.8	44.0
Mean number of hours asleep (SD)	7.3 (1.3)	7.1 (1.4)	7.3 (1.4)
**Other factors:**			
Rarely/never/sometimes happy, %	18.0	25.8	19.7
Treatment for depression, %	13.1	20.4	14.7
Mean number of children (SD)	2.3 (1.2)	2.2 (1.5)	2.3 (1.3)
Not in work, %	65.1	70.9	66.4
No participation in group activities, %	39.7	35.9	38.9
**Follow-up for IHD mortality (I20-I25) from date of hospital admission**			
Mean years of follow-up (SD)	3.7 (2.7)	3.6 (2.7)	3.7 (2.7)
IHD deaths (n)	468	215	683

[a] Percentages were calculated based on women with complete information for that specific variable.
Abbreviations: IHD, ischemic heart disease
